# Effects of two different variants in the *MAGT1* gene on B cell subsets, platelet function, and cell glycome composition

**DOI:** 10.3389/fimmu.2025.1547808

**Published:** 2025-03-18

**Authors:** Lucía del Pino Molina, Elena Monzón Manzano, Carla Gianelli, Luz Yadira Bravo Gallego, Javier Bujalance Fernández, Paula Acuña, Yolanda Soto Serrano, Keren Reche Yebra, María Bravo García-Morato, Elena Sánchez Zapardiel, Elena G. Arias-Salgado, Rebeca Rodríguez Pena, Nora Butta, Eduardo López Granados

**Affiliations:** ^1^ Center for Biomedical Network Research on Rare Diseases (CIBERER U767), Madrid, Spain; ^2^ Hematology Unit, La Paz University Hospital-IdiPAZ, Madrid, Spain; ^3^ Lymphocyte Pathophysiology in Immunodeficiencies Group, La Paz Institute for Health Research (IdiPAZ), Madrid, Spain; ^4^ Clinical Immunology Department, La Paz University Hospital, Madrid, Spain; ^5^ Research on Comprehensive Care for Transplanted Children and Adolescent Group, La Paz Institute for Health Reserach (IdiPAZ), Madrid, Spain

**Keywords:** XMEN, MAGT1, platelets, calcium influx, B cell phenotype, glycosylation

## Abstract

**Introduction:**

X-linked immunodeficiency with magnesium defect, Epstein-Barr virus (EBV) infection and neoplasia (XMEN) disease is caused by hemizygous loss of function (LOF) gene variants in *MAGT1*. MAGT1 is a plasma membrane transporter of magnesium (Mg^2+^) that plays a relevant role in immune responses and acts as a second messenger in intracellular signaling, but also it is involved in the glycosylation of proteins. Here we report two gene variants in the *MAGT1* gene from two different families with XMEN disease. A *de novo* variant c.97_98 delinsC affecting one member of one family and three members of a second family presented the hemizygous variant c.80``3G>A, p.Trp268Ter, causing a premature stop codon.

**Methods:**

We performed a functional validation of these two variants in the *MAGT1* gene and their association with decreased NKG2D expression, uncontrolled EBV viremia, and the development of lymphoma-associated complications in three members of the same family.

**Results:**

We analyzed the B-cell compartment, we found that the B-cell expansion is driven by immature/transitional (CD5^-^ and CD5^+^) and naïve B cells. The patients presented normal absolute counts of memory B-cells (MBCs) but with differences between them in the diversity of immunoglobulin heavy chain (IgH) isotype distribution in MBC, and diverse reduction of plasma cells. We also explored the alterations of platelets due to hemorrhagic events and a history of thrombocytopenia in some of our patients. We found diminished TRAP-induced calcium flux, P-selectin and CD63 exposure in XMEN patients, while when platelets from patients were stimulated ADP the results were similar to healthy controls. Finally, we explored the glycosylation pattern in platelets and lymphocytes. Our results suggest that different variants in *MAGT1* gene might result in different effects on NK cells and platelet glycome composition.

**Discussion:**

Here, we report the two different outcomes regarding EBV-driven lymphoproliferative complications, the family with three members affected that developed the malignant lymphoproliferative complications before XMEN diagnosis, and the patient with early diagnose of MAGT1 deficiency due to EBV viremia. As a recommendation, XMEN disease should be ruled out in males with impaired clearance of EBV-infection and EBV-driven lymphoproliferative complications.

## 1 Introduction

X-linked immunodeficiency with magnesium defect, Epstein-Barr virus (EBV) infection and neoplasia (XMEN) disease (OMIM: 300853) is caused by hemizygous loss of function (LOF) gene variants in *MAGT1* (1). XMEN disease is considered an inborn error of immunity (IEI) classified within the subgroup of immune dysregulation with EBV susceptibility and lymphoproliferative conditions (2). Some new evidence in glycosylation defects (3) suggests “N-linked glycosylation defect” should be added to the XMEN acronym (4).

MAGT1 is a plasma membrane transporter of magnesium (Mg^2+^), the most prevalent bivalent cation in cells (1). Mg^2+^ is an essential cofactor for adenosine triphosphate (ATP), which regulates calcium (Ca^2+^) concentrations in cells. It plays a relevant role in immune responses and acts as a second messenger in intracellular signaling (5). MAGT1 is a critical regulator of intracellular free Mg^2+^ in the immune system. LOF variants in MAGT1 lead to reduced levels of Mg^2+^and prevent transient T-cell receptor (TCR)-induced Mg^2+^ flux, essential for optimal T-cell activation (1). Low Mg^2+^ levels result in diminished expression of the receptor NKG2D, involved in natural cytotoxicity in CD8^+^ T cells and NK cells, causing inability to control EBV viremia (6). EBV primary infection usually occurs in adolescents and young adults, and turns to an asymptomatic carrier state in most cases (7). However, when the immune system, mainly through CD8^+^ and NK cells, cannot control EBV in immunocompromised patients, lymphoma risk increases, since EBV is an oncogenic virus (7, 8).

MAGT1 not only acts as an Mg^2+^ transporter, it is also a subunit of the oligosaccharyl-transferase complex (OST) (8, 9) in the endoplasmic reticulum (ER), involved in the glycosylation of proteins (9). Alterations in the glycosylation process led to incomplete glycosylation of several proteins, such as NKG2D, CD28 and CD70 associated with the TCR, and others involved in the neural function that connects directly with the progressive neurological and cognitive decline observed in some patients (8, 10). Recently, alterations in platelet function have also been described (3).

Here we report two gene variants in the *MAGT1* gene, affecting four members of two different families with XMEN disease. We performed a functional validation of these two variants in the *MAGT1* gene (11) and their association with decreased NKG2D expression, uncontrolled EBV viremia, and the development of lymphoma-associated complications in three members of the same family. We also analyzed alterations in the B-cell compartment and platelets due to hemorrhagic events and a history of thrombocytopenia in some of our patients. Finally, we explored the glycosylation patterns in platelets and lymphocytes.

## 2 Methods

### 2.1 Samples

Blood samples from patients and healthy donors (HDs) were collected at La Paz University Hospital after obtaining informed consent, according to the principles of the Declaration of Helsinki. The study received approval from the hospital Ethics Committee (PI-2833). The clinical data was taken from the clinical records obtained during routine medical visits for diagnosis and follow-up at the Clinical Immunology Department outpatient clinic.

### 2.2 Genetic analysis

Genomic DNA was isolated from the patients’ peripheral blood. Genetic analysis using a next-generation sequencing (NGS)–customized panel (12) revealed hemizygous variants in the *MAGT1* gene. A *de novo* variant c.97_98 delinsC, p.M33RfsTer12 in P1 from family one. A second family with three affected brothers (P2, P3 and P4) presented the hemizygous variant c. 803G>A, p.Trp268Ter, causing a premature stop codon at position 268 in the MAGT1 protein of 367 aminoacids. Although disease inheritance is X-linked, we could not detect the variant in DNA from the mother’s peripheral blood, saliva or nails, suggesting she is likely a gonosomal mosaic.

### 2.3 Flow cytometry analysis

#### 2.3.1 Phenotypic analysis of lymphocytes in peripheral blood

Blood samples from the patients were processed and stained with the EuroFlow 8-color *PIDOT, Pre-GC B cell* tube, and *IgH subclasses* tube (13–15) following the EuroFlow SOPs for staining, instrument set-up, and calibration, as previously described (16). The FACS Canto II cytometer (BD Biosciences) was used for data acquisition and data analysis was performed with the *Infinicyt* software (CytognosSL, Salamanca, Spain).

NKG2D plasma membrane expression was analyzed by flow cytometry. Briefly, whole blood was stained with CD3 FITC, CD16 PE, CD56 APC, CD4 V450, CD45 V500, and NKG2D PerCp Cy 5.5 (BD Biosciences), and erythrocytes were lysed with FACS Lysing Solution (BD Biosciences), washed, and acquired on an FACSCanto II flow cytometer (BD Biosciences) and DxFlex cytometer (Beckman Coulter). FlowJo software was used for the data analysis.

#### 2.3.2 Evaluation of platelet function

Human peripheral blood samples were collected in standard 3.8% sodium citrate tubes (BD, Madrid, Spain). The blood cell counts were performed with a Coulter AcT Diff cell counter (Beckman Coulter, Madrid, Spain).

Platelet-rich plasma (PRP) was obtained by whole citrated blood centrifugation (150 × g for 20 minutes at 23°C) (17). To obtain washed platelets, the top two-thirds of the PRP volume were collected and centrifuged (650 x g for 10 minutes at 23°C) after adding acid-citrate-dextrose (1:10). The pellet was resuspended in an equal volume of HEPES buffer (10 mM HEPES, 145 mM NaCl, 5 mM KCl, and 1 mM MgSO4, pH 7.4). All samples were fully analyzed within 2 hours of sampling.

To study platelet function, the PRP was diluted to 1:5 with HEPES buffer and incubated with or without 100 μmol/L thrombin receptor-activating peptide 6 (TRAP; Bachem, Switzerland) or ADP (Sigma-Aldrich, Madrid, Spain) at room temperature (RT). Following incubation, fluorescein isothiocyanate (FITC) anti-P-selectin mAb (BD Pharmingen, San Diego, California, United States), FITC anti-CD63 mAb (BD) or FITC-PAC1 (BD), a mAb that recognizes activated conformation of fibrinogen receptor, were added for 15minutes at RT. After incubation, the platelets were diluted in HEPES buffer for flow cytometry analysis.

We determined the surface expression of the fibrinogen receptor by labeling diluted PRP with phycoerythrin (PE)-mAbs against its ɑIIb (CD41, BioCytex, Marseille, France) and FITC-mAb against its β3 (CD61, BD, Madrid, Spain) subunits. Surface expression of von Willebrand factor (VWF) receptor was determined using FITC-mAbs against its CD42a and CD42b subunits (BD Pharmingen, Madrid, Spain).

### 2.4 Calcium flux in lymphocytes and platelets

Intracellular Ca^2+^ fluxes were measured using FuraRed (FuraRed, cell permeant, Thermo Fisher Scientific). An amount of 1x10^6^ PBMCs from patients and healthy donors was incubated with 1μM Fura Red-AM in loading buffer (Hank’s Balanced Salt solution medium supplemented with 5% fetal calf serum) at 30 °C for 30 min in the dark (18, 19), then stained with CD19 APC for B cells and with CD3 FITC for T cells (BD Biosciences). The cells were washed and resuspended in loading buffer at room temperature and finally warmed to 37 °C for 5 min before acquisition. Baseline intracellular Ca^2+^ levels were measured for 60 s, followed by stimulation of the B-cell receptor (BCR) with 35 μg/mL F(ab)2 anti-Human IgM (Southern Biotech) and in T cells induced with anti CD3 and anti CD28 (BD Biosciences). At the end of each Ca^2+^ measurement, the cells were stimulated with 5 μg/mL of ionomycin (Sigma) to measure maximum Ca^2+^ signaling. Data was acquired on a FACSCanto II flow cytometer (BD Biosciences) and the data analysis was performed with FlowJo software. Calcium flux experiments were run in duplicates.

PRP was obtained, as previously described (17). To obtain washed platelets, the PRP was centrifuged (650 g for 10 min at 23°C) after adding acid-citrate-dextrose (1:10). The pellet was resuspended in an equal volume of HEPES buffer. Intracellular Ca^2+^ fluxes in platelets were loaded with 1μM Fura Red in HEPES buffer at 37°C for 20 minutes in a 5% CO2 atmosphere (20). The platelets were identified by flow cytometry by forward (FSC) and sideward (SSC) light scatter properties, and also stained with CD61 FITC (BD Biosciences). Baseline intracellular Ca^2+^ levels were measured for 60 s, followed by stimulation with 100µmol/L of ADP (Sigma Aldrich) or 100 μmol/L of thrombin receptor-activating peptide 6 (TRAP, Bachem, Switzerland) at RT. The tube was removed to add ADP or TRAP and replaced immediately, to ensure data acquisition continued as soon as possible. Finally, the platelets were stimulated with 2 μg/mL ionomycin (Sigma Aldrich) to measure maximum Ca^2+^ signaling. The platelet experiments were performed in duplicate in all XMEN patients.

For the analysis of calcium flux in lymphocytes and platelets, median fluorescence intensity (MFI) was obtained for each parameter using Flow Jo software (LLC). Fura red fluorescence is emitted in the blue laser (PerCP-Cy5-5-A) and violet laser (BV-500). To obtain the calcium flux, we set the ratio of the MFI in the violet laser to the MFI in the green laser at each second of acquisition in baseline conditions and after activation with anti-IgM and ionomycin. The graphs were drawn with GraphPad Prism version 9.0 software (San Diego, CA, USA).

### 2.5 Glycosylation experiments

Glycan fluorescence intensity in lymphocyte subsets was quantified by flow cytometry, as previously described (21). Briefly, peripheral blood mononuclear cells (PBMCs) were obtained after Ficoll density gradient centrifugation (Ficoll-Paque Premium; VWR International, Eurolab). A total of 5x10^5^ PBMCs were resuspended in 100 µL of RPMI-1640 supplemented medium (10% FCS, 100 U/mL penicillium, 100 µg/mL streptomycin and 2 mM glutamine), washed, resuspended in PBS+0.5%BSA, and incubated on ice for 30 minutes. The cells were then stained with CD3 APC, CD4 PerCP, CD16 PE, CD56 PE (BD Biosciences) and CD19 PE Cy7 (Beckman Coulter) for 30 minutes on ice, washed, and then stained with FITC-labeled lectins (Vector Laboratories, Barcelona, Spain), as shown in **Supplementary Table S1**, at 4 μg/mL for 30 minutes on ice. Finally, the cells were fixed with 4% formaldehyde (Cell Signaling). The samples were acquired in DxFLEX (Beckman Coulter) and analyzed using FlowJo software (Flow-Jo LLC). Glycan fluorescence intensity was measured as the MFI for each lectin in CD4^+^ and CD8^+^T cells, B cells and NK cells.

To characterize the platelet glycome, washed platelets (50x10^3^ platelets/µL) were incubated with FITC-labeled lectins (10 µg/mL, Vector Laboratories, Barcelona, Spain), for 30 min at 37°C and analyzed by flow cytometry.

#### 2.5.1 Measurement of caspase activity of platelets

To analyze active caspase-3,7, -8, or -9, the PRP was diluted 10-fold with isotonic HEPES-buffered saline with Ca2^+^ (5 mM HEPES; 140 mM NaCl; 2.7 mMKCl; 0.42 mM NaH2PO4; 1 mM MgCl2; 2 mM CaCl2; 12mM NaHCO3; 0.35% bovine serum albumin; 5 mM dextrose, pH 7.4) containing 2 mM of Gly-Pro-Arg-Pro (Sigma-Aldrich, Madrid, Spain) to prevent fibrin formation. Next, PE-labeled anti ɑIIb mAB was added and FAM-DEVD-FMK, or FAM-LETD-FMK, or FAM-LEHD-FMK (Millipore, Madrid, Spain) was added to the samples, which were analyzed by flow cytometry.

### 2.6 Statistics

The data analysis was performed using GraphPad Prism version 9.0 software (San Diego, CA, USA). We determined the statistical differences between the patient and controls by applying the nonparametric Mann-Whitney test. The differences were considered to be statistically significant and were coded as follows: *p<0.05; **p<0.01; ***p<0.001; ****p<0.0001.

## 3 Results

### 3.1 Clinical characteristics of the patients

The principal clinical characteristics of the patients are summarized in **Table 1**. P1 is a 11-year-old male who was referred for recurrent lymphadenopathies and family history of Burkit’s lymphoma. He had three otitis media and pertussis at seven years of age. During infancy, recurrent lymphadenopathies were observed and resolved with antibiotic therapy at 3, 4, and 6 years of age. At 11 years of age, he developed a cervical lymph node enlargement associated to elevated EBV levels (range: 5230-405000 copies/mL). He was diagnosed of XMEN disease at 12 years old, and has been on supplemental magnesium since then. After six and a half years of follow-up, P1 has not recurrence of lymphadenopathy or detection of EBV levels. P1 has increased transaminase levels (range AST: 41-111UI/L, ALT: 41-81UI/L), with positive ANA 1/160, anti-smooth muscle antibodies 1/160, and anti-F-actine antibodies (weak positive), without fibroscan involvement and no need for immunosuppressive treatment. P2 is a 31-years-old male referred to our clinic for progressive decrease in serum immunoglobulins (Ig) on immunoglobulin replacement therapy (IgRT) since 23 years of age. He presented a history of three pneumonias, recurrent otitis, herpes zoster with dermatosis, and frequent gingivitis. At 18 years of age, he was diagnosed with Hodgkin lymphoma and treated with chemotherapy. He had a history of fluctuating thrombocytopenia and mild neutropenia. And at 23 years of age, he suffered an episode of generalized epilepsy. He had two healthy brothers and other two brothers that have suffered lymphoma (P3 and P4). With all this data a genetic analysis was performed revealing XMEN disease in the three affected brothers with lymphoma. All the three brothers presented a history of EBV infection and elevated transaminases levels. P3 is a 28-years old male, he was diagnosed of Hodgkin lymphoma at the age of 5 with a relapse two years later resolved with chemotherapy and radiotherapy. With 10 years old he presented a thrombocytopenia that required rituximab treatment and splenectomy. He presented neutropenia and he has suffered during his infancy of pneumonia, mastoiditis, sinusitis, and perianal abscesses. P4 was a 33-years old male that dies of refractory seizures in the context of the relapse of his lymphoma. At 24 years of age, he was diagnosed of diffused large B cell lymphoma (DLBCL) and he was treated with chemotherapy and rituximab. He presented thrombocytopenia at 4 years old; he suffered from recurrent herpes infections on his lips, with 25 years old he presented a pneumonia, and when he was referred to our clinic, he had hypogammaglobulinemia. Other relevant clinical data, immunological assessment at diagnosis including vaccination titers and Ig levels, together with treatments are detailed in **Table 1**. All the four patients started with Mg^2+^ oral supplementation after XMEN diagnosis.

**Table 1:** Principal clinical characteristics of patients.

Patient	P.1	P.2	P.3	P.4
Age of diagnosis, years	12	25	22	28
Follow up, years	6	6	6	5, death at 33 y
MAGT1 variant				
cDNA	c.97_98delinsC	c. 803G>A	c. 803G>A	c. 803G>A
Protein	p.M33RfsTer12	p.Trp268Ter	p.Trp268Ter	p.Trp268Ter
Recurrent infection				
Epstein Barr Virus	Yes (peripheral blood and cervical node)	Yes (peripheral blood)	Yes (Lymphoma tissue)	Yes (peripheral blood)
Herpes simplex virus	Yes	No	No	No
Pneumonia	No	Yes	Yes	Yes
Otitis media	Yes	Yes	Yes (with mastoiditis)	No
Varicella + recurrent zoster	No	Yes	No	Yes
Cytopenias (age onset y)				
Anemia	No	No	No	No
Lymphopenia	No	No	No	No
Neutropenia	No	Yes (mild)	Yes	No
Thrombocytopenia	No	Yes (mild and fluctuating)	Yes (5, 10 ITP)	Yes (4, ITP)
Lymphoproliferation (age onset y)	Lyphadenopathies (3)	Hodgkin Lymphoma, nodular sclerosis (18)	Hodgkin Lymphoma, mixed cellularity, (5, and 7 relapse)	Non Hodgkin Lymphoma - DLBCL (24)
Location	Cervical	Mediastinal and abdominal	Mediastinal	Mesenteric adenopathies, left femur, bilateral iliac crest; CNS
Other manifestations				
Neurological manifestations	No	Yes (Seizures)	Yes (Intelectual/developmental delay)	Yes (Behaviour abnormalities post chemotherapy)
Endocrine manifestation	No	No	No	Yes (Multinodular goiter)
Hepatic manifestation	Yes (elevated ALT/AST)	Yes (Hepatomegaly, elevated ALT/AST)	Yes (elevated ALT/AST)	Yes (elevated ALT/AST)
Immunological assessment (at diagnosis)				
Tetanus toxoid (post-vaccination response)	Yes	n.d*	Yes	Yes
Pneumoccoccal (post-vaccination response)	No	n.d*	Yes	Yes
IgG, mg/dL	564	778*	609	477
IgA, mg/dL	118	<8	112	72
IgM, mg/dL	237	43	96	119
IgE, IU/mL	15.9	6.64	<2	<2
Treatments (age y)				
Rituximab	No	No	Yes (10, ITP)	Yes (24, DLBCL)
Splenectomy	No	No	Yes (10, ITP)	No
IgRT	No	Yes (23)	No	No
Magnesium supplementation	Yes (12)	Yes (25)	Yes (23)	Yes (29)

Age (y=years); ITP, immune thrombocytopenia; DLBCL, diffuse large B cell lymphoma; ALT, alanine transaminase; AST, aspartate transaminase; IgRT, immunoglobulin replacement therapy; n.d, not determined *because the patient was on IgRT.

### 3.2 Phenotypic analysis of lymphocytes in peripheral blood

**Figure 1:**
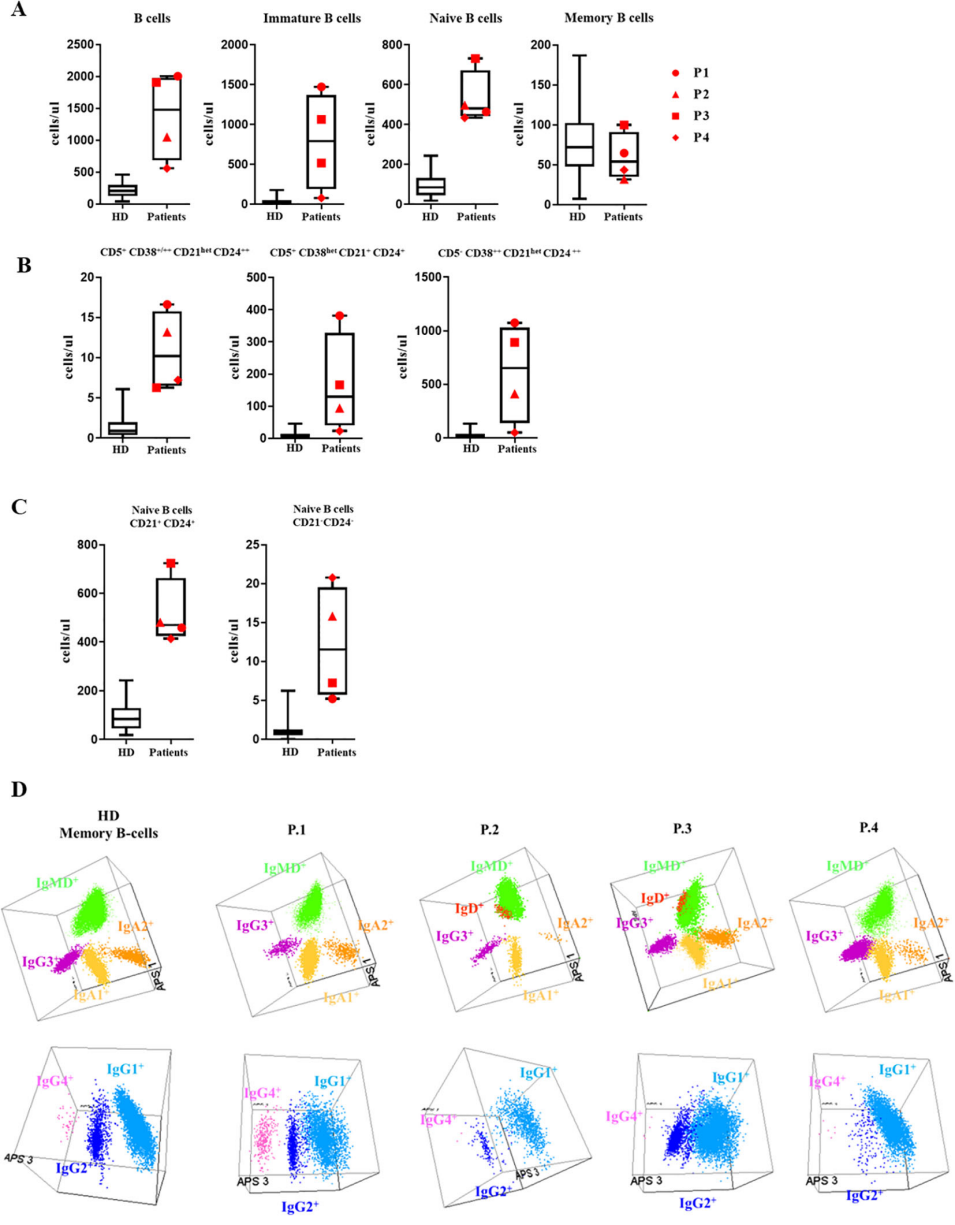
B-cell subsets: **(A)** B-cell subset distribution: Absolute counts of total B cells and the major subsets of immature, naive and memory B cells (MBC) are shown using box and whisker plots separately for HD and XMEN patients P1 (red circle), P2 (red triangle), P3 (red square), and P4 (red diamond). **(B)** Absolute counts of CD5^-^ CD38^++^ CD21^het^ CD24^+++^, CD5^+^ CD38^+/++^ CD21^het^ CD24^++^ and CD5^+^ CD38^het^ CD21^+^ CD24^+^ immature B-cells. **(C)** Absolute counts of CD21^+^CD24^+^ and CD21^-^CD24^-^ naïve B-cell subset counts in XMEN vs HD. **(D)** The results of the immunoglobulin heavy chain IgH (IgH) isotype distribution in memory B cells (MBC) analyzed by flow cytometry in the patients and a representative healthy donor (HD). Representation on a balanced 3-dimension (3D) automated population separator (APS) diagram, constructed using the first three principal components (PC1 to PC3) derived from PC analysis (PCA) performed with the *Infinicyt* software.

In all the XMEN patients we observed greatly increased absolute numbers of B cells compared to the reference normal values in healthy controls (22) (**Figure 1A**), except in P4, where the value was closer to the normal range (**Table 2**). The higher quantities of B cells were due to the expansion of the subsets from the pre-germinal center (GC) compartment immature/transitional B cells and naïve B cells (**Figure 1A**). A more detailed analysis of the pre–GC B-cell compartment showed the expansion of CD5^+^ CD38^+/++^ CD21^het^ CD24^++^, CD5^+^ CD38^het^ CD21^+^ CD24^+^and CD5^-^ CD38^++^ CD21^het^ CD24^++^ immature/transitional B lymphocytes for patients P1, P2 and P3 (**Figure 1B**. Regarding the mature naive B-cell subset, an increase in CD21^+^ CD24^+^, together with the expansion of CD21^-^ CD24^-^ naive B-cell counts were present in all XMEN patients, except for P1 (**Figure 1C**). Total absolute counts of memory B-cells (MBCs) were within the normal range in all XMEN patients (**Figure 1A**, nonetheless we further dissected the diversity in this compartment by analyzing the immunoglobulin heavy chain (IgH) isotype distribution in MBC (**Figure 1D**) and plasma cells compared to the normal reference values per age group previously described (15). In the MBC compartment, P1 had a normal distribution of IgH subclasses for his age. In family 2, P2 and P4 had lower IgG2 (0,72 and 0,34 cells/μL respectively vs. normal reference values for their age 1,6-30 cells/μL) and IgA2 MBC (0,10 and 0,5 cells/μL respectively vs. normal reference values for their age 1.2-18 cells/μL), while the IgG4 MBC was absent in P2 and P3. Within the plasma cells distribution, P1 presented normal total plasma cells counts for his age, mostly IgM and IgA1, but undetectable IgG isotypes and low IgA2 plasma cells (0.24 cells/μL vs. normal reference range 0.3-36 cells/μL). P2 had the most severe reduction (0.31 cells/μL vs. 1.1-25 cells/μL, normal reference values) to the extent that only IgM plasma cells were evidenced. P4 had reduced plasma cells for his age (0.73 cells/μL vs. 1.1-25 cells/μL, normal reference values for their age) undetectable IgG isotypes and low IgA1 (0.28 cells/μL vs. 0.3-6.9 cells/μL) and IgA2 plasma cells (0.06 cells/μL vs. 0.2-4.2 cells/μL). However, P3 conserved almost normal absolute counts of plasma cells (6.28 cells/μL vs. 1.1-25 cells/μL) with normal distribution of IgH subclasses except for the absence of IgG3 and IgG4. Notably, P3 had higher numbers of IgD^+^-only MBC (5.9 cells/μL vs. normal reference values <0.01-2.4 cells/μL) and plasma cells than the HDs.

**Table 2:** Distribution in percentages and absolute values of different subsets of peripheral blood B-cells in XMEN patients.

	P1 (%)	P1 Absolute numbers	Reference absolute numbers (10-17 y)	P2 (%)	P2 Absolute numbers	P3 (%)	P3 Absolute numbers	P4 (%)	P4 Absolute numbers	Reference absolute numbers (18-39 y)
B-cells	32.4	2378	360 (174-630)	16.7	1033.73	27.4	1904.4	13.2	559.68	220 (41-470)
Pre GC B cell compartment			Reference absolute numbers							Reference absolute numbers
Immature/Transitional B-cells	73.49	1473	27 (5,7-126)	49.4	517	55.9	1065	14.2	79	27 (5,7-126)
CD5-CD38++CD21hetCD24+++	0.83	17	0,89 (0,13-4)	1.3	13	0.3	6	1.3	7	0,89 (0,13-4)
CD5+CD38+/++CD21het CD24++	19.05	382	5,60 (1,1-35)	8.9	94	8.7	166	4.0	23	5,60 (1,1-35)
CD5+CD38+CD21+CD24+/++	53.61	1074	17 (4-94)	39.2	410	46.9	892	8.9	50	17 (4-94)
Naive B cells	23.18	465	85 (24-203)	47.5	497	38.4	731	77.7	435	85 (24-203)
CD21+CD24+	22.89	459	84 (23-201)	46.0	481	38.0	724	73.9	414	84 (23-201)
CD21-CD24-	0.26	5.2	0,74 (0,02-3,7)	1.5	16	0.4	7	3.7	21	0,74 (0,02-3,7)
CD21-CD24++	0.03	0.6	0,4 (0-0,14)	0.02	0	0.02	0	0.08	0	0,4 (0-0,14)
Post GC B cell compartment	P1 (%)	P1 Absolute numbers	Reference absolute numbers (10-17 y)	P2 (%)	P2 Absolute numbers	P3 (%)	P3 Absolute numbers	P4 (%)	P4 Absolute numbers	Reference absolute numbers (18-39 y)
Memory B-cells	7.76	184.5	68 (31-160)	3.58	37.01	5.65	107.60	8.92	49.92	91 (23-221)
Memory IgMD+ B cells	4.87	115.8	29 (17-78)	1.71	17.68	1.4	26.66	2.72	15.22	38 (7,9-122)
Other Memory B-cells	0	0.0		0.8	8.27	1.18	22.47	1.87	10.47	
Memory IgG1+ B cells	0.93	22.1	18 (7-42)	0.55	5.69	0.79	15.04	1.82	10.19	18 (3,2-40)
Memory IgG2+ B cells	0.27	6.4	3 (0,7-10)	0.07	0.72	0.24	4.57	0.06	0.34	5,9 (1,6-30)
Memory IgG3+ B cells	0.11	2.6	3 (1,1-8,3)	0.12	1.24	0.16	3.05	1.34	7.50	3 (0,5-8,4)
Memory IgG4+ B cells	0.1	2.4	0,2 (<0,01-2,9)	0	0.00	0	0.00	0.01	0.06	0,4 (<0,01-2,4)
Memory IgA1+ B cells	1.29	30.7	9 (2,9-21)	0.29	3.00	1.43	27.23	1.01	5.65	11 (2,1-43)
Memory IgA2+ B cells	0.14	3.3	2,7 (0,8-5,9)	0.01	0.10	0.14	2.67	0.09	0.50	4,1 (1,2-18)
Only IgD+	0.03	0.7	0,2 (<0,01-2,4)	0.03	0.31	0.31	5.90		0.00	0,2 (<0,01-2,4)
Normal PC	0.18	4.3	4,4 (1,3-27)	0.03	0.31	0.33	6.28	0.13	0.73	4,4 (1,1-25)
Other Normal PC		0.0		0.01	0.10	0.02	0.38	0.02	0.11	
PC IgM+	0.07	1.7	0,8 (0,2-5,7)	0.01	0.10	0.02	0.38	0.05	0.28	0,4 (0,05-4,7)
PC IgA1+	0.08	1.9	3,1 (0,5-14)	0.01	0.10	0.16	3.05	0.05	0.28	1,7 (0,3-6,9)
PC IgA2+	0.01	0.2	1 (0,3-3,6)	0	0.00	0.01	0.19	0.01	0.06	0,7 (0,2-4,2)
PC IgG1+	0	0.0	1,1 (0,1-4,8)	0	0.00	0.01	0.19	0	0.00	0,4 (0,05-4,4)
PC IgG2+	0	0.0	0,5 (0,08-0,8)	0	0.00	0.02	0.38	0	0.00	0,2 (<0,01-2,6)
PC IgG3+	0	0.0	0,03 (<0,01-0,4)	0	0.00	0	0.00	0	0.00	0,03 (<0,01-0,3)
PC IgG4+	0.01	0.2	<0,01 (<0,01-0,2)	0	0.00	0	0.00	0	0.00	<0,01 (<0,01-0,4)
PC IgD+	n.d	n.d		n.d	n.d	0.09	1.71	n.d	n.d	

Percentages of total B cells are referred to total leukocytes, while the percentages of different B cells subsets are referred to total B cells. Reference absolute numbers are expressed as median absolute numbers of cells (range). The reference absolute numbers from healthy controls in the Pre GC B cell compartment are from del Pino Molina et al., 2021 (reference 22). The reference values per age group in the Post GC B cell compartment are from Blanco et al., 2018 (reference 15); n.d, not detected.

No other relevant alterations were found in the lymphocyte populations, except for a slight increase in the CD4/CD8 ratio in P2, P3 and P4, with absolute numbers of CD4^+^ T cells within the normal range (**Table 3**). Lymphocyte proliferation in response to anti-CD3 was also tested during clinical routine follow up and analyzed by using tritiated thymidine uptake. Indeed, P1 and P2 showed normal proliferation assay while P3 and P4 showed reduced lymphocyte proliferation in response to anti-CD3.

**Table 3:** Distribution in percentages and absolute values of different T cells subsets and NK cells of peripheral blood in XMEN patients obtained from PIDOT tube.

	P1 (%)	P1 Absolute numbers	Reference absolute numbers (10-17 y)	P2 (%)	P2 Absolute numbers	P3 (%)	P3 Absolute numbers	P4 (%)	P4 Absolute numbers	Reference absolute numbers (18-29 y)
T cells	35.7	2903	(930-3477)	33.8	2092	37.6	2594	39.3	1667	(564-2935)
CD4+CD8-	20.6	1675	(576-1891)	10	618	14.3	989	14.3	606	(207-1900)
Naive (CD27+CD45RA+)	13.2	1075	(264-1484)	5.7	355	6.4	442	8.6	365	(74-1173)
Central memory/transitional memory (CD27+CD45RA-)	7.2	583	(244-593)	4	248	7.3	503	5.1	214	(117-886)
Effector memory (CD27-CD45RA-)	0.18	14.9	(27-222)	0.23	14	0.59	40.6	0.49	20.8	(14-500)
Terminally differentiated (CD27-CD45RA+)	0.018	1.4	(0-46)	0.019	1.2	0.056	3.8	0.15	6.2	(0-87)
CD8+CD4-	11.2	917	(261-1189)	14.7	907	19.8	1366	19.4	824	(160-1103)
Naive (CD27+CD45RA+)	6.6	541	(94-986)	4.2	260	4.4	306	7.7	325	(33-737)
Central memory/transitional memory (CD27+CD45RA-)	4	325	(76-427)	10	620	9.4	652	10.6	447	(54-422)
Effector memory (CD27-CD45RA-)	0.14	11.3	(6-62)	0.093	5.8	0.13	8.7	0.13	5.6	(5-69)
Terminally differentiated (CD27-CD45RA+)	0.32	26.4	(3-93)	0.27	16.4	5.6	387	0.9	38.4	(0-144)
Terminally differentiated (CD27int-CD45RA+)	0.17	14	(3-284)	0.07	4.4	0.17	11.7	0.19	8.1	(1-240)
CD4+CD8+	0.032			0.19	11.8	0.086	5.9	0.41	17.4	
CD4-CD8-/dim TCRgd+	1.7	155	(8-53)	8	494	2.5	171	4.2	179	(11-470)
CD4-CD8-/dim TCRgd-	2.1	153	(56-332)	0.99	61.6	0.9	62.4	0.95	40.2	(5-79)
NK cells	4.7	384	(109-1021)	4.7	292	2.7	188	4.5	189	(81-615)

Percentages of T cells subsets and NK cells are referred to total leukocytes. Reference values for absolute counts of peripheral blood T cells and NK cells calculated with the Euroflow PID orientation tube in healthy controls per age group from van der Burg et al., 2019 (reference 14).

### 3.3 NKG2D surface expression in lymphocytes

**Figure 2:**
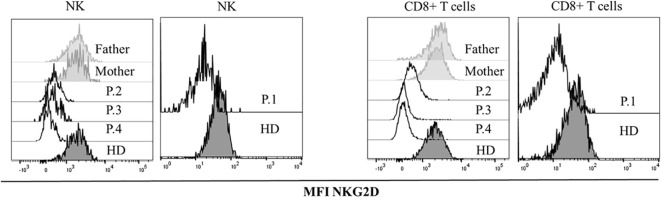
NKG2D expression. Histograms showing median fluorescence intensity (MFI) of NKG2D in healthy donors (HDs), healthy parents and patients with the variants in *MAGT1*gene, in CD8^+^ and NK lymphocytes.

Affected patients had severely reduced NKG2D expression in CD8 T cells and NK cells compared to the healthy controls and their healthy parents (**Figure 2**).

### 3.4 Calcium flux in lymphocytes and platelets

**Figure 3:**
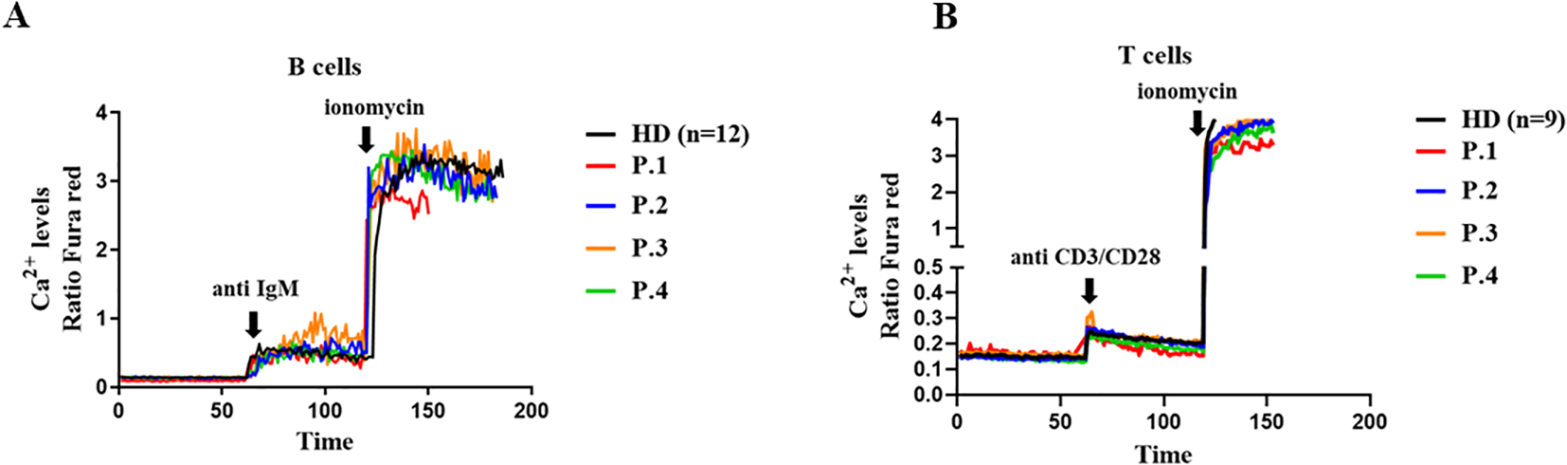
Calcium flux in lymphocytes. A Cytosolic calcium ion (Ca^2+^) levels at baseline and induced by anti-IgM and ionomycin in B cells **(A)** and in T cells induced with anti CD3/CD28 **(B)** from XMEN patients and healthy donors (HD). The results from the patients are from two independent experiments.

The gene variants found in these XMEN patients did not impact on antigen-receptor induced Ca^2+^ influx upon anti-IgM stimulation in the patient’s B cells or in anti CD3/CD28 T cells. A similar slope of Ca^2+^ influx was evidenced when compared with HDs, reaching as well a similar Ca^2+^influx when stimulated with ionomycin as the maximum positive control, similar to the HDs (**Figures 3A, B**).

**Figure 4:**
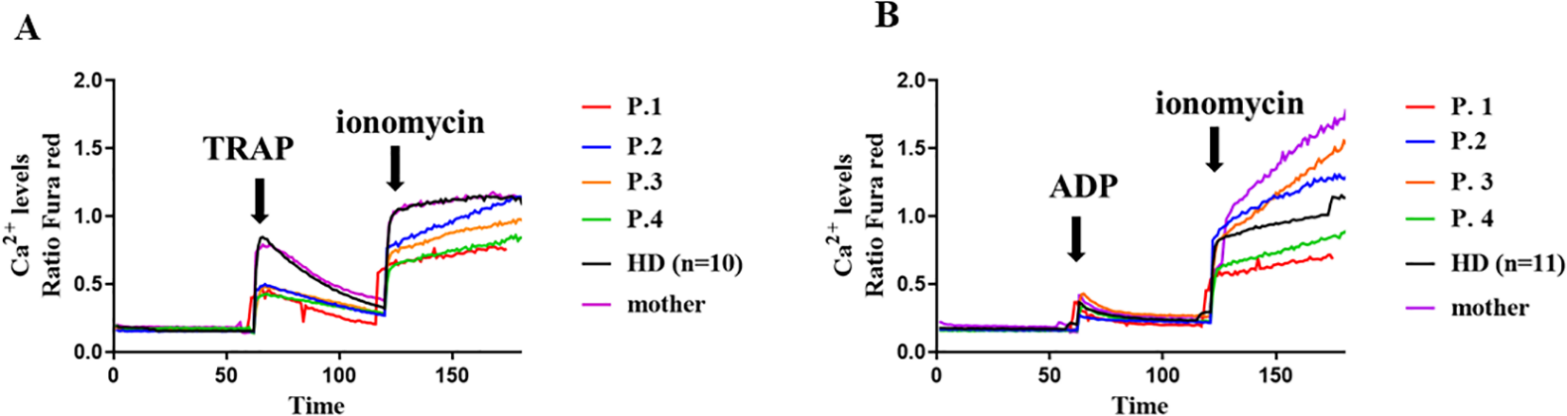
Calcium flux in platelets. Cytosolic calcium ion (Ca^2+^) levels at baseline and induced by TRAP **(A)** and ADP **(B)** in platelets from the XMEN patients and healthy donors (HD) (n=10). Flow cytometry analyses of platelets from XMEN patients were performed at least twice on different days.

The presence of thrombocytopenia in some patients prompted us to investigate the functionality of platelets. To do this, we analyzed baseline calcium flux and after stimulation with TRAP and ADP. The variants c.97_98delinsC and p.Trp268Ter in XMEN decreases Ca^2+^ influx on TRAP stimulation in the patient’s platelets compared with the HDs (**Figure 4A**). However, ADP stimulation in XMEN patients resulted in calcium levels comparable with those of HDs (**Figure 4B**). For both stimulants, platelets from the mother of family two showed similar results to the HDs.

### 3.5 Functional assays on platelets

**Figure 5:**
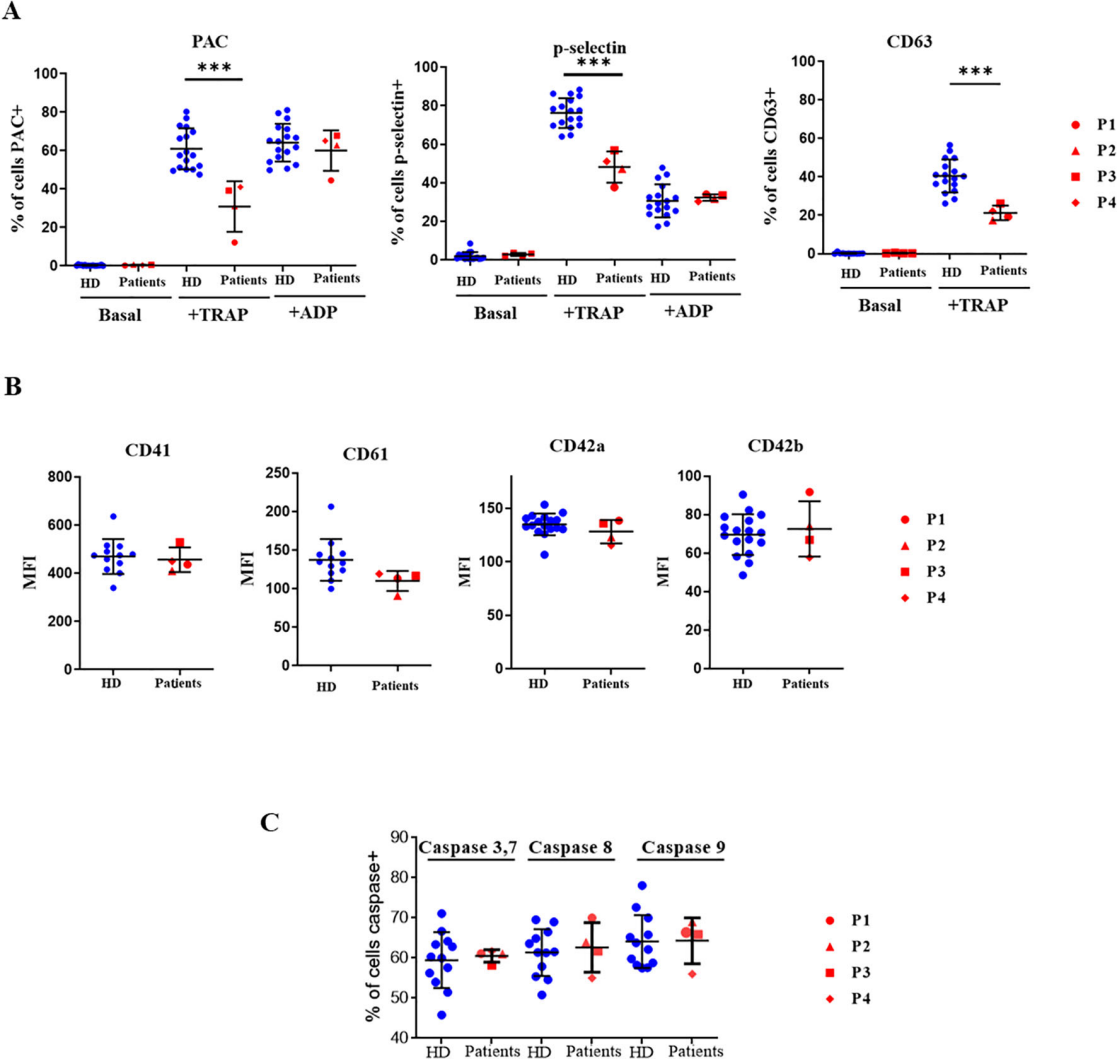
Functional assays in platelets. Platelets from healthy controls and XMEN patients analyzed by flow cytometry. Patient’s samples are processed in two independent experiments. **(A)** Platelet activation markers evaluated under baseline conditions or after stimulation with 100 µM TRAP or 20 µM ADP and incubation with fluorescein isothiocyanate (FITC)-PAC1, FITC-anti-P-selectin mAb or FITC-anti-P-CD63 mAb. Data are expressed as the % of positive cells. **(B)** Fibrinogen receptor subunits CD41 and CD61 and von Willebrand factor subunits CD42a and CD42b determined in resting platelets with the respective specific mAbs labeled with FITC, the results are expressed as mean fluorescence intensity (MFI). **(C)** Caspase activities in quiescent platelets from controls and XMEN patients expressed as the % of positive cells. ***p<0.001.

To evaluate whether diminished TRAP-induced calcium flux affected platelet function, platelet activation markers were determined in platelets stimulated by 100 µM TRAP or 20 µM ADP. As shown in **Figure 5A**, TRAP-induced fibrinogen receptor activation and P-selectin and CD63 exposure were lower in XMEN patients. As observed in ADP-induced calcium flux the two variants studied in XMEN patients did not affect the ability of platelets to be activated when ADP was used as an agonist. Moreover, impairment to platelet TRAP-induced stimulation capacity was not due to a lower content of either fibrinogen receptor or vWF receptor on their surface (**Figure 5B**). It has been reported that increased apoptosis leads to decreased platelet activation (17) but this does not appear to be the reason for the diminished response to TRAP because caspase activities in platelets from XMEN patients were similar to those from healthy controls (**Figure 5C**).

### 3.6 Glycosylation in lymphocytes and platelets

Since *MAGT1*-deficient patients have a defect in glycosylation, we studied the exposure of glycoside residues on the surface of lymphocytes and platelets.

**Figure 6:**
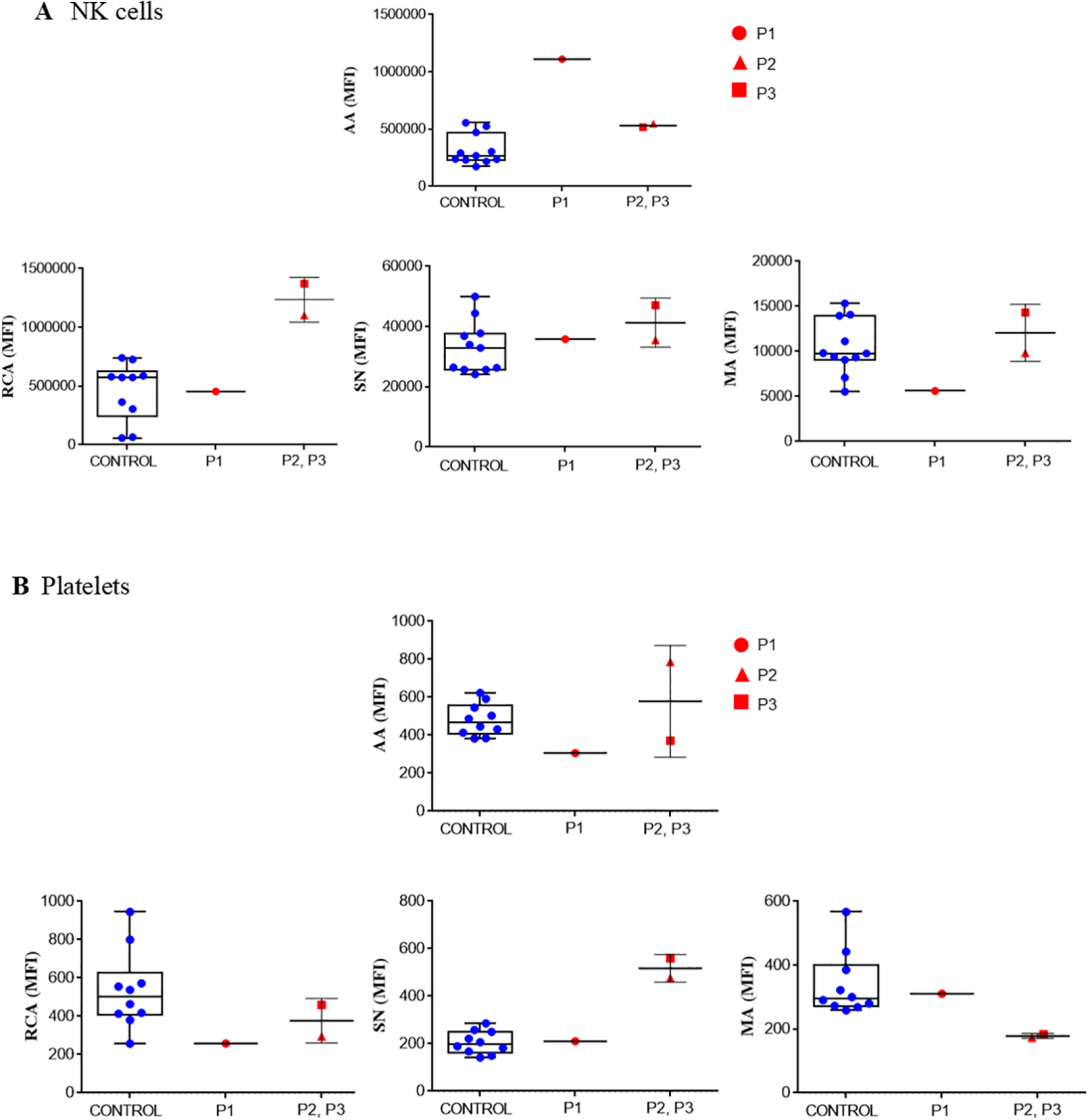
Glycosylation. Lectin binding determined in NK cells **(A)** and in resting, washed platelets **(B)** from healthy control and XMEN patients P1 (red circle), P2 (red triangle) and P3 (red square). The following lectins were tested: FITC-Aleuria aurantia (AA), FITC-Ricinus communis agglutinin (RCA), FITC-Sambuca nigra agglutinin (SN) and FITC-Maackia amurensis (MA). Samples were analyzed by flow cytometry analysis. Data are expressed as mean fluorescence intensity (MFI).

As observed in **Figures 6A, B**, the degree of exposure of certain glycoside residues on NK cells and platelets differed from that observed in NK cells from healthy controls and depended on the type of variant in the *MAGT1* gene. On NK cells (but not on platelets), the c.97_98delinsC mutation (P1) induced higher exposure of α1,6-fucose and less sialic acid, even when the proportion of sialic acid attached to terminal galactose in α2-3 and α26 linkages was similar to that seen in healthy controls.

Platelets from patients P2 and P3 with the variant c. 803G>A, p.Trp268Ter exposed α1,6-fucose and sialic acid within the normal range, but sialic acid attached to terminal galactose was lower in the α2-3 linkage and higher in the α2-6 linkage than the pattern observed in platelets from healthy controls. It has been previously reported that changes in the platelet glycome may be due to an increase in apoptosis. However, this does not seem to be the case because activity of caspases 3,7, 8 and 9 in platelets from our patients were within the range observed in platelets from healthy donors (data not shown).

## 4 Discussion

MAGT1 is a plasma membrane transporter of Mg^2+^, an ion critical for a number of biological processes, including immune responses and platelet regulation. Several ion channelopathies are associated with immune defects (23), being mutations and deletions in Mg^2+^ transporters that are more severe than mutations in Ca^2+^ transporters (5). Different animal models have been used to explore its function. In zebra fish, abolished expression of *MAGT1* is lethal (5), while in knockout mice with MAGT1 deficiency present fewer T cells, but without reduced NKG2D expression. Similarities between MAGT1 deficiency in mice and humans are related to B-cell expansion (24); however, in our patients at least, the expansion was in the pre-GC while in mice it is in the GC B-cell compartment and plasma cells. Previous studies in XMEN patients mostly focused on the T-cell compartment, defect cytotoxicity in CD8^+^ T cells and NK cells, causing the incapacity to control EBV viremia (7). Here, we explored in depth the alterations in the B-cell compartment and platelets of four male patients with MAGT1 deficiency.

B-cell expansion is a common feature reported in XMEN cases (4, 25). MAGT1 variants could impose a disturbed intracellular signaling. Specifically, MAGT1 mouse models presented B-cell expansion with increased phosphorylation of proteins downstream BCR signaling, leading to higher PKC activation and calcium flux. Together that causes the accumulation of marginal zone B cells and increased plasmablast differentiation (24). Moreover, MAGT1 deficiency causes the impairment of control EBV infection in resting B cells, that induces a transcriptional reprogramming leading to a continuous entry, progression and egress from GC, proliferation and terminal B-cell differentiation toward plasmablasts (26, 27). Our comprehensive analysis of the B-cell compartment with the *Pre-GC B cell* tube allowed us to perform a standardized panel in which detailed analysis of the immature/naïve B cell compartment. In 3/4 XMEN patients, we evidenced high expansion of immature B cells based on CD38, CD5, CD24 and CD21 expression. We found that the B-cell expansion is driven by immature/transitional (CD5^-^ and CD5^+^) and naïve B cells. Rowane et al, reported a XMEN patient with predominance of CD5^+^ B cells (28), also in X-linked lymphoproliferative disease there is an expansion of immature/transitional B cells (29). In two of our patients, we also identified an expansion of IgD^+^-only memory B cells. Lymph node biopsies in XMEN patients also revealed atypical B-cell population co-expressing CD5 (4) and in another case peripheral blood expansion of B-cell IgD^+^ was observed (10). While low total memory B cells have also been extensively reported (7, 25, 30), this is the first time to our knowledge that a complete memory and plasma B-cell isotype distribution has been performed. Hypogammaglobulinemia have been extensively reported in MAGT1 deficiency patients (10). The glycosylation defects in XMEN disease have also impact on humoral immunity. It has been evidenced that the reduced glycosylation of several proteins, including the Ig heavy chain, and particularly IgA heavy chain, impacts on its stability reducing its secretion (8, 31). We found differences between the siblings with a history of lymphoma and rituximab treatment with lower diversity of IgH MBC, while the children with XMEN disease and EBV viremia but no malignant proliferation had an adequate B-cell distribution for their age, except for the lower plasma cell counts. In P3 and P4 the hypogammaglobulinemia could be inherent to the genetic disorder and sustained by the rituximab treatment, since impairment of B-cell maturation is extensively reported in post-rituximab patients.

The variant p. Trp268Ter in the family with three affected brothers (P2, P3, P4) who developed malignant lymphoproliferative complications before diagnosis was reported in a young Chinese boy, with EBV infection and other recurrent upper respiratory tract infections (32). The early diagnosis of XMEN disease in this patient may have prompted him to develop malignant complications.

We also observed a reduction of NKG2D expression in NK cells and CD8^+^T cells, as reported in other studies (33). It has been shown that NKG2D is under-glycosylated in one XMEN patient and in CRISPR-Cas9-engineered HEK293 WT cells, depleted for STT3B and MAGT1, which lowers the steady-state levels of the protein (9, 34). In addition to the under glycosylation of NKG2D, the glycosylation defects have affect several immune-response proteins, in particular CD28, which strongly amplifies TCR signaling upon antigen recognition, as well as in other proteins implicated in the effector functions of T cells such as HLA-DR and CD70 (8). In particular, the genetic deficiency of CD70 predisposes to impaired control of EBV infection (10). The defects in glycosylation also affect innate immunity, for example of PERL protein that is present in saliva and airway secretions and generates antimicrobial components (8).

Epistaxis and delayed bleeding after surgery have been reported in some patients, independently of the degree of thrombocytopenia (33, 35). These hemorrhagic manifestations may be due to a lack of platelet activation capacity. Mg^2+^ is well buffered in the cytoplasm of healthy human platelets, which is 533.2 ± 12.2 µmol/L under baseline conditions (36), and MAGT1 has been proposed as playing a key role in its regulation. In MAGT1-deficiency patients, we observed that TRAP-induced fibrinogen receptor activation and P-selectin and CD63 exposure were lower. This is consistent with the lower Ca^2+^ flux observed in platelets from XMEN patients exposed to the same stimulus. Platelet activation impairment was not caused by a reduction in their fibrinogen receptors. On the other hand, the responses of platelets from XMEN patients to ADP stimulation (activation of the fibrinogen receptor, release of alpha-granule contents, and induced Ca^2+^ flux) did not differ from those of platelets from healthy controls. These differences between response to TRAP and ADP stimulation may be because these agonists induce different intracellular mechanisms of platelet activation (37). A differential sensitivity of these pathways to MAGT1 dysfunction, either in its role as a magnesium channel or as a mediator of protein glycosylation, could explain why only the response to platelet PAR1 and PAR4 receptor activation was affected and not the response mediated by purinergic receptors. In addition, changes in the glycosylation of thrombin and purinergic receptors may alter their ability to bind the agonist and to specifically couple to different G protein subtypes (38, 39). Similar to our study, Kauskot et al. (3) observed lower platelet aggregation, fibrinogen receptor activation, and Ca^2+^ mobilization induced by PAR-1 receptor activation with either thrombin or TRAP. However, these authors also found a lower platelet response to ADP. Such differences may be methodological, as those authors used washed platelets whereas we used diluted PRP, and perhaps the degree of platelet activation by ADP, which is considered a mild platelet agonist (40), may differ depending on the experimental conditions of the flow cytometry assays, altering the sensitivity of the assay. However, our flow cytometry results are supported by the observation that there is no change in ADP-induced calcium flux. Regarding calcium flux in lymphocytes, we have detected normal calcium flux in PBMCs stimulated with anti IgM or TCR agonist such as CD3/CD28, in B and T cells respectively, as previously reported (1).

Impaired platelet function is consistent with the fact that protein glycosylation is a post-translational modification critical for normal platelet function (41, 42). Most platelet proteins are glycosylated in megakaryocytes, but it has been shown that the latter can package Golgi elements into vesicles with glycosyltransferases and transport them to nascent platelets. In addition, platelets contain sufficient levels of donor sugar nucleotides, suggesting that they could provide glycosylation of *de novo* translated glycoproteins (43).

MAGT1 was identified as a non-catalytic subunit of the oligosaccharyltransferase (OST) complex. Each OST complex contains several accessory subunits non-covalently associated with an enzymatic subunit (STT3A and STT3B). It facilitates N-glycosylation by transferring mannose oligosaccharides to specific asparagine (N) residues in the protein sequence (8, 44). Platelets have structural N-glycan diversity, such as abundant high-mannose N-glycans, particularly Man5, and diverse sialylated di-, tri-, and tetra-antennary complex N-glycans, including polylactosamine-bearing structures and antennal fucosylation (45). Flow cytometry analyzing the binding of lectins, carbohydrate-binding proteins with relative specificity for particular glycans (see **Supplementary Table S1**), was used to study glycoside residues exposed on the platelet surface. Our results suggest that different variant in *MAGT1* gene might result in different effects on platelet glycome composition. For example, both variants described in our patients exposed less α-linked N-acetylgalactosamine and did not seem to reduce sialic acid exposure. Nevertheless, variant p. Trp268Ter appeared to induced more sialic acid attached to terminal galactose in α-2,6 linkage and less in α-2,3 linkage (binding of SN and MA respectively). The family with the three affected brothers that we studied had thrombocytopenia. This observation may be explained by the fact that gene variants associated with loss of function in MAGT1 are recognized as congenital disorders of glycosylation that can cause thrombocytopenia (41). XMEN can therefore be considered a congenital disorder of glycosylation that presents as a combined immune deficiency with platelet dysfunction (46). As changes in the composition of the platelet glycome have been associated with increased apoptosis (17), we investigated caspase activity in platelets from XMEN patients. We did not find any differences in the activities observed in platelets from healthy controls, perhaps because apoptosis is associated with loss of sialic acid, which was not the case in our patients.

Given the especially high risk in bone marrow transplantation (BMT) of hemorrhagic events due to platelet alterations (35) and unsuccessful magnesium supplementation therapy (47), there are several strategies for correcting XMEN disease. Brault et al. propose a cell therapy based on MAGT1 mRNA correction in T and NK cells (48). In addition, attempts using gene therapy with CRISPR/Cas9 and adeno-associated vectors (AVV) have been made to restore NKG2D expression and glycosylation function, by correcting both CD8 and NK cells to combat infections rapidly and provide a long-term response by editing hematopoietic stem cells (HSPC) (49).

As a recommendation, XMEN disease should be ruled out in males with impaired clearance of EBV-infection and EBV-driven lymphoproliferative complications. Furthermore, new studies are needed to help clarify the neurological deterioration observed in some patients.

## Data Availability

The raw data supporting the conclusions of this article will be made available by the authors, without undue reservation.
