# Supplementary material for: Effects of two different variants in the MAGT1 gene on B cell subsets, platelet function, and cell glycome composition
Source: Front Immunol. 2025 Mar 18;16:1547808. doi: 10.3389/fimmu.2025.1547808 (PMC11958192; doi:10.3389/fimmu.2025.1547808)
Supplement: Supplementary file 1 [file Table1.pdf]

Supplementary Table S1: Lectins used in flow cytometry experiments and their glycoside binding specificity.

Lectin	Aleuria aurantia	Maackia amurensis	Ricinus communis agglutinin I	Sambuca nigra agglutinin
Abbreviation	AA	MA	RCA	SN
Sugar specificity	α1,6-Fucose	sialic acid attached to terminal galactose in α-2,3 linkage	Galactose<br>GalNAc	sialic acid attached to terminal galactose in α-2,6 linkage

GalNAc: N- acetylgalactosamine; β-GlcNAc: N- acetylglucosamine.
